# Topical vancomycin powder does not affect patella cartilage degeneration in primary total knee arthroplasty and conversion rate for secondary patella resurfacing

**DOI:** 10.1007/s00402-022-04721-w

**Published:** 2022-12-20

**Authors:** Benjamin Jacob, Georgi Wassilew, Rüdiger von Eisenhart-Rothe, Steffen Brodt, Georg Matziolis

**Affiliations:** 1 Orthopaedic Department Waldkliniken Eisenberg, University Hospital Jena, Campus Eisenberg, Klosterlausnitzer Straße 81, 07607 Eisenberg, Germany; 2 Department of Orthopaedic Surgery, University Hospital Greifswald, Greifswald, Germany; 3 Department of Orthopaedics and Sports Orthopaedics, Klinikum rechts der Isar, Technische Universität München, Munich, Germany

**Keywords:** Vancomycin, TKA, SSI, PJI, Cartilage, Patella resurfacing, Toxicity

## Abstract

**Introduction:**

Vancomycin powder (VP) is an antibiotic first introduced in pediatric spinal surgery to prevent surgical site infections (SSI). Recently its topical application was expanded to total hip and knee arthroplasty (THA, TKA) and anterior cruciate ligament reconstruction (ACLR). Toxicity to cartilage is the subject of current research. The aim of this study was to prove the hypothesis that topical application of VP in TKA does not result in a degeneration of patella cartilage. We propagate that the conversion rate for secondary patella resurfacing is not influenced by its use.

**Materials and methods:**

Between 2014 and 2021, 4292 joints were included in this monocentric retrospective cohort study. All patients underwent TKA without primary patella resurfacing. After a change of the procedure in the hospital, one group (VPG) was administered VP intraoperatively. The other group (nVPG) received no VP during surgery (nVPG). The remaining perioperative procedure was constant over the investigation period. Conversion rates for secondary patella resurfacing for both groups were determined without making distinctions in the indication. A second cohort was composed of patients presenting for follow-up examination 12 months after TKA and included 210 joints. Retrospective radiographic evaluations were performed preoperatively, before discharge and at follow-up examination. Patella axial radiographs were analyzed for patella tracking (lateral patellar tilt, patellar displacement) and patella degeneration (Sperner classification, patellofemoral joint space).

**Results:**

There was no significant difference in the conversion rate for secondary patella resurfacing (4.24% VPG, 4.97% nVPG). Patella tracking and patella degeneration did not differ significantly between both groups.

**Conclusions:**

The topical application of VP does not influence the conversion rate for secondary patella resurfacing. Moreover, it does not result in a degeneration of patella cartilage in TK.

**Level of evidence:**

Retrospective case series, Level III.

## Introduction

Periprosthetic joint infection (PJI) and surgical site infection (SSI) represent a disastrous complication in orthopedic surgery. Despite the supposedly low incidence of 1–2%, consequences for the patient including multiple follow-up operations with the unsatisfying functional outcome are significant [1, 15, 16, 25]. Rates of revision of any cause of up to 40.2% within two years after primary arthroplasty were described [9].

The topical application of vancomycin powder (VP) was established in pediatric spinal surgery more than 10 years ago. The efficiency of reducing SSI has been well documented by numerous studies [4, 13, 20, 29]. In the meantime, VP was introduced to total hip and knee arthroplasty (THA, TKA) and anterior cruciate ligament reconstruction [7, 14, 18, 21]. Although it remains an object of current research and debate, first results to combat PJI and SSI appear to be promising [11, 22].

Vancomycin is an antibiotic agent belonging to the group of glycopeptides. The bactericidal effect is based on the inhibition of cell-wall synthesis. Precursors required for the cross-linking of murein cannot be used because of the inhibition of the transglycosylation reaction [23].

With increasing widespread application possibilities of VP, possible toxic effects on human tissue should be clarified before general recommendations for the prophylaxis of infections can be made.

Dose- und time-dependent chondrotoxicity of VP could already be demonstrated in multiple in vitro investigations [2, 24, 27]. It is still not clear whether this effect is diminished in vivo*.* The aim of this study was to investigate possible degenerating tissue effects of the intraarticular application of VP in a clinical setting. Therefore, we chose a high-volume TKA model without primary patella resurfacing where native cartilage remains preserved. We hypothesized that topical VP has no influence on patella cartilage und therefore does not increase the rate of secondary patella resurfacing after TKA.

## Materials and methods

This is a monocentric retrospective cohort study. It was approved by the local ethics committee. Resulting from the retrospective and anonymized study design, an informed consent was not necessary according to the ethics committee approval. However, all patients included agreed to the topical use of vancomycin as an off-label use. All methods were carried out in accordance with the relevant guidelines and regulations based on the approval. Inclusion criterion was primary TKA for the treatment of osteoarthritis of the knee. Exclusion criteria were revision TKA and contraindications for the topical use of vancomycin (i.e. allergies to glycopeptides). Between 2014 and 2021 all patients receiving a primary cemented posterior stabilized TKA without patella resurfacing for the treatment of osteoarthritis of the knee were included (Smith and Nephew Legion, DePuy Attune fixed bearing, Bbraun e.motion, Waldemar Link Gemini). After an internal change of procedure at the hospital, VP (Vancomycin HEXAL^®^ 1,0 g, Hexal AG, Germany, Holzkirchen) was administered intraarticularly (VPG) before capsule closure. Before that time point, VP was not administered at all (nVPG). It was not used selectively so that an inclusion bias due to restricted use in specific indications could be excluded. The remaining perioperative procedure was constant over the investigation period.

Out of these two groups, cases receiving a secondary patella resurfacing were registered. Indication for secondary patella resurfacing was persistent anterior knee pain (> 6 months) and a scintigraphically “hot patella” or radiographical complete loss of joint space between patella and trochlea. These indication criteria did not change during the study period.

A second cohort was composed of patients routinely presenting for follow-up examination 12 months after TKA in our outpatient clinic between 2014 and 2021. This subgroup was analyzed retrospectively and represents a negative selection of the basic population. Within this cohort, there was no distinction between the groups receiving vancomycin (VPG) or not (nVPG). Epidemiological data (age, sex, side) and radiographic data (patellar displacement, patellar tilt, patellofemoral joint space, Sperner classification) were collected preoperatively (one day prior to surgery), before discharge (five days after surgery) and at follow-up examination [28].

**Fig. 1:**
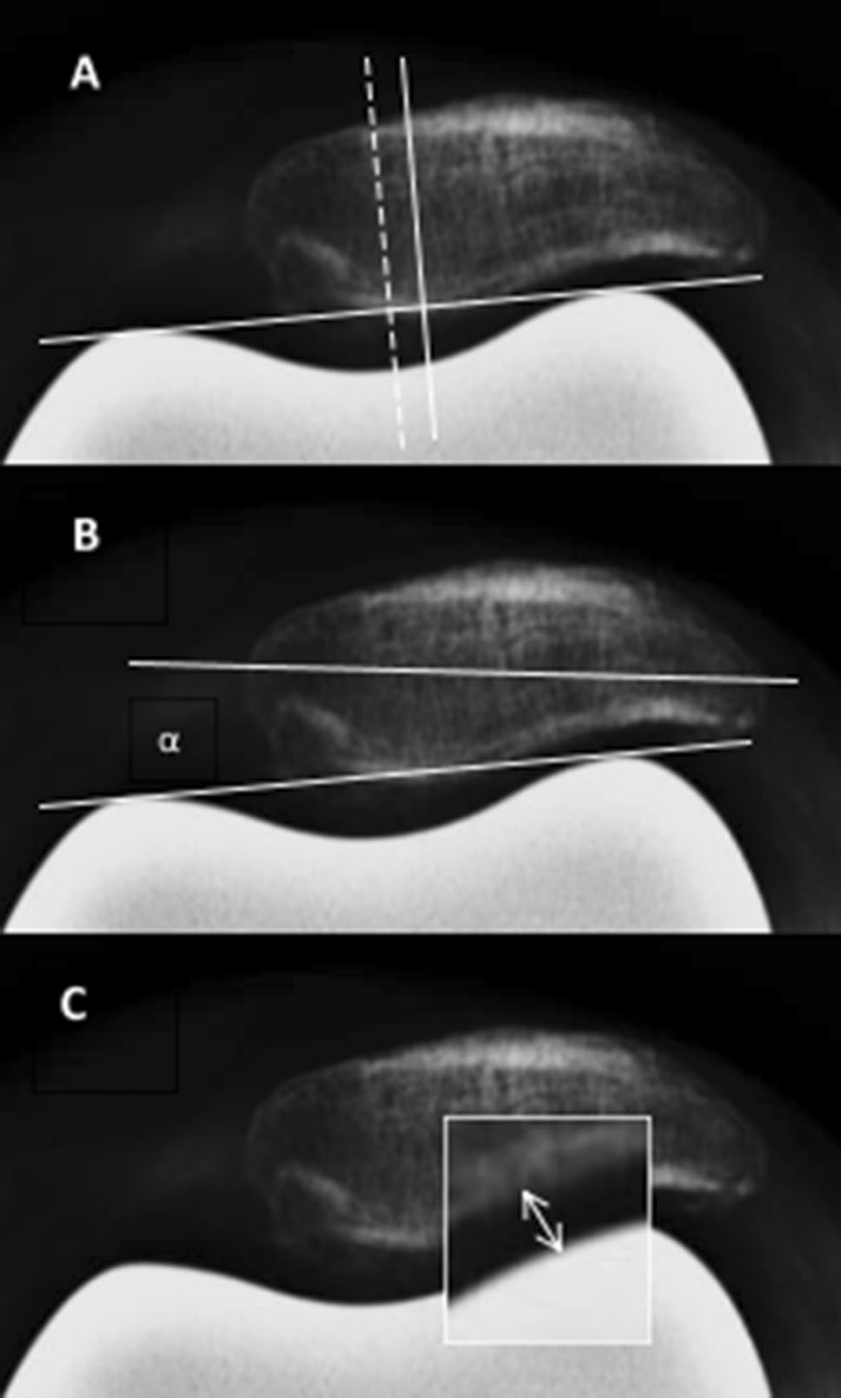
measurement techniques: **A** patellar displacement, **B** patellar tilt, **C** patellofemoral joint space

Patella axial radiographs (60° flexion) were analyzed to determine patella tracking and patella degeneration. AGFA Impax (Agfa-Gevaert N.V., Mortsel, Belgium) and ImageJ were used as digital radiology imaging systems. Patellar displacement is the distance between the line through the center of the femoral condyles and the line through the center of the patella (Fig. 1A) [12]. Patellar tilt is the angle between the patella width line and the line from the anterior limits of the femoral condyles (Fig. 1B) [12]. We defined the patellofemoral joint space as the minimum distance between the articular facet of the patella and the surface of the femoral component of the prosthesis (Fig. 1C). Evaluation of the patellofemoral joint (PFJ) degeneration was performed according to the classification by Sperner (Table 1) [28].

**Table 1:** Sperner classification [28]

Grade	
0	No degenerative changes
I	Definitive subchondral sclerosis with minimal osteophytes on the patella
II	Definite osteophytes on the patella
III	Narrowing of patellofemoral joint space, osteophytes on the patella
IV	Tight joint space and large osteophytes with a deformed patella

### Statistical analysis

Statistical analysis was performed using Microsoft Office Excel (Microsoft Corporation, Redmont, Washington, USA) applying Student’s paired *t*-test, *χ*^2^ test and Mann–Wilcoxon–Whitney *U* test. Normal distribution was tested using the Kolmogorov–Smirnov test. A *p* value of < 0.05 was considered as statistically significant.

## Results

Over 8 years, 4292 joints were included in the study. Secondary patella resurfacing was performed in 196 cases (4.57%). Intraarticular VP was used in 1931 joints (VPG) and was not used in 2361 joints (nVPG). Demographic data showed no statistically significant differences between both groups (Table 2a). Conversion rate for secondary patella resurfacing was 4.24% (100 cases) in the VPG and 4.97% (96 cases) in the nVPG. There is no statistically significant difference (*p* = 0.25, Table 2b).

**Table 2:** **a** Patient demographics, **b** secondary patella resurfacing

	VPG	nVPG	*p* value
**a**			
Number	2361	1931	
Age	66.21	66.86	0.10
Male:female	1053:1308	835:1096	0.19
Right side:left side	1171:1190	975:956	0.72
**b**			
Patella resurfacing	100 (4.24%)	96 (4.97%)	0.25
No patella resurfacing	2261	1835	

The second cohort consisted of 210 joints (129 VPG, 81 nVPG). Medium age was 63.4 years (VPG), respectively 64.9 years (nVPG, Table 3a). Further demographic data showed no statistically significant differences between both groups (Table 3a). Patella tracking measurements showed no significant differences at all observation periods (Table 3b). Average patellar displacement was 1.03 mm to lateral (preop.), 0.10 mm to medial (discharge) and 0.97 mm to lateral (follow-up) for the VPG, resp. 1.03 to lateral, 0.46 mm to medial and 0.82 mm to lateral for the nVPG. The average patellar tilt was 7.10° (preop.), 5.54° (discharge) and 8.11° (follow-up) for the VPG, resp. 6.77°, 5.64° and 8.94° for the nVPG. No significant difference in patella degeneration for both groups could be identified (Table 3c). Patellofemoral joint space decreased by 1.13 mm within the VPG and by 0.91 mm within the nVPG during one year (*p* = 0.13). Sperner score decreased by 0.28 points in both groups (*p* = 0.47) [28].

**Table 3:** **a** Patient demographics, **b** patella tracking preoperatively, postoperatively and at follow-up, **c** radiographic patella degeneration: the differences between measurements early postoperative and follow up are given

	VPG	nVPG	*p* value
**a**			
Number	129	81	
Male:female	60:69	34:47	0.52
Age (years)	63.4	64.9	0.15
Right side:left side	67:62	43:38	0.56
**b**	preoperative		
Patellar displacement (mm)	1.03 ± 3.56 lateral	1.03 ± 2.55 lateral	0.50
Lateral patellar tilt (°)	7.10 ± 5.05	6.77 ± 4.03	0.33
	postoperative		
Patellar displacement (mm)	0.10 ± 3.61 medial	0.46 ± 3.62 medial	0.24
Lateral patellar tilt (°)	5.54 ± 4.75	5.64 ± 4.95	0.43
	follow-up		
Patellar displacement (mm)	0.94 ± 3.82 lateral	0.82 ± 6.13 lateral	0.43
Lateral patellar tilt (°)	8.11 ± 4.93	8.94 ± 8.05	0.18
**c**			
Patellofemoral joint space (mm)	1.13 ± 1.54	0.91 ± 0.96	0.13
Sperner score (pts.) [28]	0.28 ± 0.45	0.28 ± 0.53	0.47

## Discussion

The main result of this study is that the topical application of VP does not influence the unresurfaced patella in TKA. VP does not affect the conversion rate for secondary patella resurfacing or radiographically measurable patella cartilage thickness.

Regardless of VP application, degeneration of patellar cartilage (Sperner classification, patellofemoral joint space) for both groups was detected during observation time [28]. These findings can be confirmed by Sato et al. who measured a reduction in the thickness of patellar cartilage to less than half within 5 years after no patellar resurfacing TKA using a ceramic femoral component and MRI [26].

We are aware that patella tracking is influenced by various parameters. Gasparini et al. summarized several factors increasing the risk for patellar maltracking in TKA: preoperative valgus; patellofemoral dysplasia; surgical approach; Q angle; tightness of lateral retinaculum; patella height/thickness; design, alignment, rotation and size of the components [8]. Bauer et al. analyzed registry data and biomechanical testing comparing posterior-stabilized (PS) and cruciate-retaining (CR) prosthesis. They found out that a multifactorial cause (increased rollback, greater external tilt, increasing facet pressure, lower quadriceps force, patellar pressure) might be responsible for a higher rate of secondary patella resurfacing regarding PS systems [5]. However, since patella tracking measurements (tilt, displacement) were comparable in both groups of our study, we believe that component positioning and implant design can be ruled out as confounding variables.

The main advantage of this investigation, despite its monocentric design, is the high number of cases included. Influencing factors relevant to TKA outcome (e.g. implant design, surgical technique, perioperative management) were constant over this study period so that the use of intraarticular vancomycin remains the only variable.

According to McConaghy et al. the optimal management of the patella during TKA remains controversial. Studies comparing primary resurfacing and non-resurfacing of the patella have reported inconsistent findings which indicates the need for further prospective randomized research [19]. Concerning our study, cases with primary patella resurfacing were excluded and the indication for secondary resurfacing was consistent between both groups.

To the best of our knowledge, there is no study examining the chondrotoxicity of topical vancomycin in vivo up to date. In vitro experiments tend to show higher cell toxicity resulting from direct chondrocyte contact without a protective three-dimensional extracellular matrix. Vancomycin powder in vitro does not have to pass barriers such as the cartilage matrix. Röhner et al., Shaw et al. and Antoci et al. could demonstrate chondrotoxicity of VP in vitro [2, 24, 27]. Liu et al. discovered significant inhibition of cell survival and cell migration (human osteoblasts, myoblasts and fibroblasts) after continuous exposure for 48 h to vancomycin in vitro [17]. The in vitro study of Chu et al. suggested that vancomycin has toxic effects on mesenchymal stem cells [6]. Atherton et al. postulated that Vancomycin did not significantly alter the molecular structure of the hamstring graft in their in vitro cell culture and ex vivo tissue experiments [3]. Ultimately Han et al. established a bone defect rat model and could demonstrate that local delivery of vancomycin may have detrimental effects on bone regeneration [10]. In a recent extensive study Wei et al. aimed to explore the efficacy and safety of intraarticular VP in the prophylaxis of infection after TKA in a rat model. General status, serum biomarkers, radiology, microbiological assay, and histopathological tests were assessed. In summary bacterial counts, knee width, tissue inflammation and osteolysis were reduced in the groups receiving intraarticular VP [30]. However, it should be critically noted, that it cannot be distinguished whether detrimental effects on tissue are a result of bacterial (MRSA) or antibiotic action.

The main limitation of the present study is its retrospective study design, which, even if all potential influencing factors remain constant, cannot exclude unknown confounders. Further studies are required before a general statement about the toxicity of topical vancomycin to cartilage in vivo can be made.

## Data Availability

The data that support the findings of this study are available on request from the corresponding author.

## Declarations

### Transparency declarations

None to declare.
